# Privacy-Preserving Task Offloading Strategies in MEC

**DOI:** 10.3390/s23010095

**Published:** 2022-12-22

**Authors:** Haijian Yu, Jing Liu, Chunjie Hu, Ziqi Zhu

**Affiliations:** 1 College of Computer Science and Technology, Wuhan University of Science and Technology, Wuhan 430065, China; 2 Hubei Province Key Laboratory of Intelligent Information Processing and Real-Time Industrial System, Wuhan 430065, China

**Keywords:** association privacy, location privacy, mobile edge computing, privacy protection, task offloading

## Abstract

In mobile edge computing (MEC), mobile devices can choose to offload their tasks to edge servers for execution, thereby effectively reducing the completion time of tasks and energy consumption of mobile devices. However, most of the data transfer brought by offloading relies on wireless communication technology, making the private information of mobile devices vulnerable to eavesdropping and monitoring. Privacy leakage, especially the location and association privacies, can pose a significant risk to users of mobile devices. Therefore, protecting the privacy of mobile devices during task offloading is important and cannot be ignored. This paper considers both location privacy and association privacy of mobile devices during task offloading in MEC and targets to reduce the leakage of location and association privacy while minimizing the average completion time of tasks. To achieve these goals, we design a privacy-preserving task offloading scheme to protect location privacy and association privacy. The scheme is mainly divided into two parts. First, we adopt a proxy forwarding mechanism to protect the location privacy of mobile devices from being leaked. Second, we select the proxy server and edge server for each task that needs to be offloaded. In the proxy server selection policy, we make a choice based on the location information of proxy servers, to reduce the leakage risk of location privacy. In the edge server selection strategy, we consider the privacy conflict between tasks, the computing ability, and location of edge servers, to reduce the leakage risk of association privacy plus the average completion time of tasks as much as possible. Simulated experimental results demonstrate that our scheme is effective in protecting the location privacy and association privacy of mobile devices and reducing the average completion time of tasks compared with the-state-of-art techniques.

## 1. Introduction

In recent years, mobile devices such as smartphones and tablets have gained popularity. According to Ericsson’s latest report, the number of mobile subscriptions worldwide has exploded and will reach 9.2 billion in 2028 [1]. The growing popularity of mobile devices has spawned new applications such as online games, virtual reality, image recognition, etc. These nascent applications often have high demands on computing resources and latency. However, the computation and storage capabilities of existing mobile devices often struggle to meet such demands [2]. The emergence of MEC has effectively solved the problem of insufficient computation and storage capacity of mobile devices. MEC, as an extension of cloud computing, has computation and storage located closer to the users of mobile devices at the edge of the network, thus, overcoming the problem of high latency in cloud computing [3,4,5,6]. By using task offloading technology, mobile devices can effectively reduce their energy consumption and completion time [4].

However, wireless transmission-based task offloading in edge computing technologies is vulnerable to eavesdropping and monitoring. Attackers can infer users’ location and usage patterns according to offloading preferences of their mobile devices and the channel quality during data transmission through edge servers. What is more, attackers can infer a user’s motion trajectory by obtaining real-time location information from multiple edge servers. As a result, there is a risk of location privacy leakage from mobile devices during task offloading. Location information is important privacy information for users and poses a huge risk if it is leaked. For example, in the field of wildlife protection, hunters can obtain the location information of wild animals if mobile devices carried by animals transmit related data to edge servers; in the field of smart transportation, attackers can get the life pattern and behavior habits of users based on their action trajectories via edge servers [7]. Meanwhile, since MEC is an open computing system, it does not have a comprehensive, complex, and highly reliable security system like traditional cloud computing. Thus, edge servers in MEC are relatively untrustworthy compared to servers in cloud computing.

In addition, tasks may have associated privacy information which refers to information with associated privacy data. When the associated privacy data is leaked separately, it will not cause the risk of privacy leakage to users. Nevertheless, when all or part of the associated privacy data is leaked, it will cause the risk of privacy leakage to users and then lead to serious consequences. For example, username and password belong to associated privacy information. When a malicious person only knows the username or password of a user, there is no threat to the user, but if the malicious person attains both of them, it will pose a threat to the user. When there is associated information between tasks and a mobile device offloads these tasks to an untrustworthy server, attackers can benefit from this associated privacy information by harming users’ interests. Tasks with associated privacy information are considered to have privacy conflicts. If there exist privacy conflicts between two tasks, offloading them to the same edge server may cause leakage of association privacy [8]. For instance, when three tasks separately containing the identity card number, cell phone number, and bank account number of a user are offloaded to the same edge server, attackers can gain these three numbers from the server, utilize them to get the password of the bank account, and steal the user’s property. Therefore, protecting the location and association privacy of mobile devices is quite important, and, therefore, a high amount of attention is paid to it.

In view of the high attention paid to the topic of privacy protection of task offloading in MEC, many researchers have studied it. In [9,10,11,12], authors have studied the location privacy problem for task offloading in MEC. In [13,14,15,16,17], authors have investigated how to reduce the risk of association privacy leakage by reducing the privacy conflicts of tasks. In [18,19], based on the concept of information entropy, authors have quantified data privacy to avoid the leakage of data privacy. In [20,21,22], authors have applied encrypting, blurring location information, and adding noise to achieve location privacy protection, which imposes significant extra overhead on mobile devices. As far as we know, no work has considered both location privacy and association privacy during task offloading.

In this paper, we consider both location privacy and association privacy of mobile devices when they offload tasks to edge servers in MEC, aiming to reduce the location and association privacy leakage as well as minimize the average completion time of tasks. We are committed to finding a scheme that adequately protects the location and association privacy of mobile devices while avoiding significant extra overhead. To achieve this goal, we design a privacy-preserving task offloading algorithm. In the algorithm, we first introduce a Mobile Support System (MSS) to protect the location privacy of mobile devices. MSS is designed to protect the location privacy of mobile devices without sacrificing performance and includes two kinds of servers: edge servers and proxy servers. Mobile devices do not communicate with the edge server directly but through the proxy server. The edge server only knows the location of the proxy server but is not aware of the real location of mobile devices [23]. Then, we select the proxy and edge servers for each task to be offloaded. We make choices based on the proxy servers’ location information in the proxy server selection policy to reduce the risk of location privacy leakage. In the edge server selection strategy, we consider the privacy conflict between tasks, the computing ability of edge servers, and the location of edge servers, to reduce the risk of association privacy leakage of mobile devices and the average completion time of tasks. Simulated experiments show that the risk of leakage for location and association privacy is effectively reduced by our designed algorithm. Moreover, the average completion time of tasks is reduced.

Our main contributions are as follows.

- To our best knowledge, we are the first to comprehensively consider the location privacy and association privacy issues of mobile devices during task offloading in MEC.
- We solve the problem of location privacy of mobile devices based on the MSS proxy forwarding technology.
- We design a privacy-preserving task offloading algorithm to protect location privacy and association privacy while minimizing the average completion time of tasks.
- Simulated experiments demonstrate the effectiveness and efficiency of our proposed algorithm.

The residual organization of this article is as follows. Section 2 outlines the related research. Section 3 establishes all models and the problem addressed in this paper. Section 4 introduces our proposed algorithm. Section 5 makes a performance evaluation of our proposed algorithm by considerable simulation experiments. Section 6 concludes the paper.

## 2. Related Research

In recent years, there have been many studies on task offloading in MEC. Most of the researchers worked on reducing time, energy consumption, and cost. In [24], Chen et al. minimized latency and energy consumption of mobile devices from a user-centric view to improve the quality of experience(QoE) in MEC. In [25], Labidi et al. stated the problem of task offloading in small cell base stations by jointly optimizing radio resources and offloading decisions to minimize energy consumption with tolerable delays. In [26], Guo et al. designed a sub-optimization algorithm with the goal of minimizing task execution time and energy consumption. In [27], Mao et al. presented a low-complexity online offloading algorithm that can significantly reduce the task execution cost in a green MEC system with an energy harvesting device. In [28], Zhou and Jadoon reduced the task execution time by rational allocation of bandwidth resources and moderate task offloading ratio. In [29], Liu et al. studied the stochastic task scheduling optimization problem in MEC from two timescales and adopted a one-dimensional search algorithm to minimize the average delay. None of the above work takes privacy into consideration.

Since MEC is an open computing system, edge servers can be provided by enterprises, governments, individuals, etc. Therefore, the reliability and security of edge servers are much less than traditional cloud servers. Privacy protection in MEC has become an important issue that cannot be ignored. Several researchers have studied privacy protection issues during task offloading. In [9,10,11,12], the authors studied the location privacy protection of mobile devices in MEC, considering the risk posed to the mobile device by location information leakage. In [9], Wang et al. considered the risk of location information leakage measured by the distance threshold of a user from the server, and defined a total cost as the combination of migration cost, user-perceived delay, and the leakage risk of location information as well as constructed an efficient privacy-preserving offloading algorithm to find an optimal solution to minimize the total cost in the long run. In [10], Li et al. adopted a task offloading scheme for privacy-preserving and device management. The scheme can obtain near-optimal latency and energy performance while preserving users’ location privacy and usage pattern privacy. In [11], a privacy-preserving secure offloading scheme was designed to minimize energy consumption, in which location privacy, usage pattern privacy, and secure transmission against eavesdroppers are considered in combination. In [12], Zhang et al. designed a novel and effective location privacy protection scheme for MEC devices, where authors make it impossible for an attacker to identify a user’s actual location by adding noise.

In [13,14,15,16,17], privacy conflicts between tasks were mainly studied to reduce the risk of association privacy leakage. In [13], Peng et al. studied the task offloading problem for VR applications with privacy protection. A privacy-aware task offloading approach based on a multi-objective optimization genetic algorithm was proposed, taking the privacy conflicts between virtual reality (VR) tasks into consideration. In [14], authors formally analyzed privacy conflicts of tasks in vehicular networks and adopted a MEC-based offloading method for vehicular network computation, which reduces the privacy conflicts between tasks, consequently degrading the risk of association privacy leakage. In [15], Liu et al. formulated an optimization problem to improve load balancing and reduce the energy consumption of all edge nodes while considering privacy conflicts and time overhead. Then, an energy-efficient task offloading method with privacy protection was proposed for privacy conflicts between tasks. In [16], Xu et al. presented an IoT-oriented privacy-preserving offloading method, considering privacy conflicts between tasks. In [17], Ma and Mashayekhy offered a privacy-designed offloading solution for vehicular edge computing considering privacy conflicts between tasks, to meet the delay requirements and reduce the energy consumption of vehicles.

Although research related to privacy protection in MEC has already existed, none of it has considered both location privacy and association privacy. Moreover, most of the studies on location privacy are based on encryption, blurring location information, adding noise, etc., which can bring the high extra cost to mobile devices. Different from the above research, both location privacy and association privacy during task offloading based on MEC are considered in our work. We use the proxy forwarding mechanism to protect the location privacy of mobile devices and figure out appropriate rules to select the proxy servers and the edge servers for task offloading, to prevent the location and association privacy of the mobile device from leaking while minimizing the average completion time of tasks.

## 3. Models and Problem Definition

In this section, the models used and the problem studied will be introduced.

### 3.1. System Model

**Figure 1:**
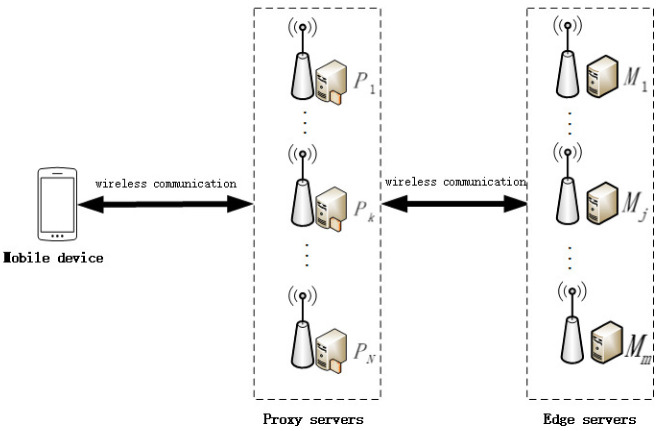
An example of a MEC system with privacy protection.

As shown in Figure 1, a single-user offloading scenario is designed, which consists of three types of entities: edge server, proxy server, and mobile device. The specific description of each entity is as follows.

**Edge servers:** edge servers provide computation and storage resources for tasks offloaded by mobile devices. These edge servers are considered untrustworthy.

**Proxy Servers:** thanks to the ubiquitous edge servers, some edge servers can be used as MSS proxy servers. These proxy servers are only responsible for forwarding tasks. Proxy servers are equivalent to virtual routers. Each proxy server can host multiple proxies, and one proxy can only serve one task at a time. Edge servers are numerous and widely distributed. Therefore, using edge servers as proxy servers can effectively reduce extra time overhead and make it easy to find the most appropriate proxy server for task forwarding.

**Mobile device:** the mobile device deploys the MSS client, which manages all incoming and outgoing traffic. It chooses to offload its tasks to the target edge server through the proxy server, so the edge server only knows the location of the proxy server, not the real location of the mobile device.

We assume that there is a mobile device *u*, *N* proxy servers, and *m* edge servers in our scenario. These devices, including the mobile device and all the servers, are randomly distributed in a two-dimensional plane with dimension 1500 m × 1500 m, and two-dimensional distances are treated as their geographical distances. The set of proxy servers and edge servers are denoted as P={1,2,3,⋯,N} and M={1,2,3,⋯,m}, respectively. The mobile device has *n* tasks and the set of tasks is denoted as t={1,2,3,⋯,n}. The attributes of task *i* can be defined as $task_{i}=\left\{d_{i},c_{i},T_{i}^{max}\right\}$, where $d_{i}$ is the data size of task *i* (bits), $c_{i}$ is denoted as the total number of CPU cycles for accomplishing the task *i*, $T_{i}^{max}$ is the maximum deadline for task *i*. Each task cannot be divided. To protect the location privacy of the mobile device during task offloading, communication between the mobile device and edge servers requires a proxy server. $\alpha_{i}$ denotes the execution position of task *i*, where $\alpha_{i}\in \left\{0,1,2,3,\cdots ,m\right\}$. when task *i* is executed locally, $\alpha_{i}=0$. When task *i* is executed at the edge server *j*, $\alpha_{i}=j$, j∈{1,2,3,⋯,m}. $\beta_{i}$ denotes the proxy server selection policy for task *i*, $\beta_{i}\in \left\{0,1,2,\cdots ,N\right\}$. When the task is performed locally, the choice of proxy is not required, at this point, $\beta_{i}=0,\alpha_{i}=0$. When task *i* is offloaded, it must be forwarded through a proxy server *j*, at this point $\beta_{i}\in \left\{1,2,3,\cdots ,N\right\}$.

### 3.2. Privacy Model

The leakage of privacy information poses a great risk to users, and privacy protection of task offloading as an important issue in MEC cannot be ignored. Here, both location privacy and association privacy during task offloading are considered.

To avoid the location information leakage of mobile devices, a proxy forwarding policy is used. In the policy, a mobile device does not communicate with an edge server directly, but through a proxy server. As a result, the edge server only knows the location of the proxy server but does not get the real location of the mobile device. According to [30], when the mobile device *u* offloads task *i* to edge server *j* via proxy server *k*, its location privacy denoted by $\lambda_{i}^{k,j}$ can be expressed as

(1) $$\begin{array}{c} \lambda_{i}^{k,j}=\frac{dis(u,P_{k})}{dis(u,M_{j})}. \end{array}$$

where $P_{k}$ is the proxy server *k* and $M_{j}$ is the edge server *j*. dis(a,b) is the distance between two devices *a* and *b*, which is calculated as follows:

(2) $$\begin{array}{c} dis\left(a,b\right)=\sqrt{{\left(a_{x}-b_{x}\right)}^{2}+{\left(a_{y}-b_{y}\right)}^{2}}. \end{array}$$

$a_{x}$ is the position of device *a* in the x-axis direction and $a_{y}$ is the position of device *a* in the y-axis direction. $b_{x}$ is the position of device *b* in the x-axis direction and $b_{y}$ is the position of device *b* in the y-axis direction. However, in [30], no specific task offloading scenario is considered. In fact, not all tasks need to be offloaded to edge servers for execution. Therefore, location privacy needs to be redefined. When the mobile device offloads task *i* to edge server *j* via proxy server *k*, the mobile device’s location privacy is computed as

(3) $$\lambda_{i}^{k,j}=\left\{\begin{array}{c} 0,\;\;\;\mathrm{if}\;\alpha_{i}=0;\\ \frac{dis(u,M_{j})}{dis(u,P_{k})},\mathrm{if}\;\alpha_{i}\neq 0. \end{array}\right.$$

A smaller value of $\lambda_{i}^{k,j}$ indicates that the mobile device is at less risk of a location privacy breach when offloading task *i* to an edge server and vice versa. Then the location privacy of the mobile device *u* can be expressed as

(4) $$\lambda =\frac{\sum_{i}^{n}\left(\lambda_{i}^{k,j}\right)}{n}.$$

At the same time, association privacy is also an important part that cannot be ignored.

**Privacy conflict:** A privacy conflict exists between two tasks if there is correlated privacy information between them and an attacker can infer some private information about the user of a mobile device based on the information from these two tasks, and harm the user. For example, when three tasks separately containing the identity card number, cell phone number, and bank account number of a user are offloaded to the same edge server, attackers can gain these three numbers from the server, utilize them to get the password of the bank account, and steal the user’s property. When task *i* and task *j* have a privacy conflict, offloading them to the same edge server will cause association privacy leakage. Privacy conflicts between tasks are considered to minimize the risk of association privacy leakage.

**Figure 2:**
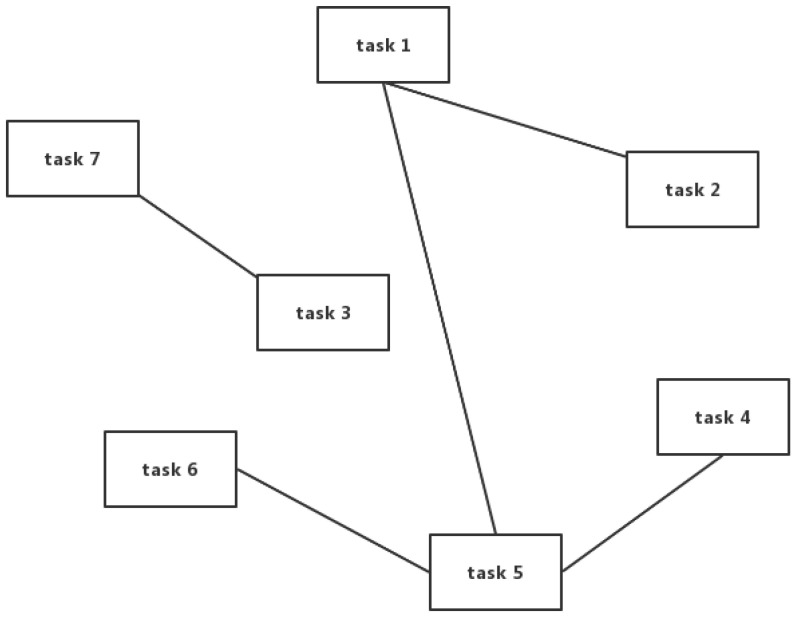
Example: privacy conflict graph with seven tasks.

**Privacy conflict graph:** We can construct a privacy conflict graph for all tasks of a mobile device. In the privacy conflict graph, each vertex represents a task; If there is a connecting line between two tasks, it means that there exists a privacy conflict between these two tasks. Figure 2 is an example of a privacy conflict graph. We can see that task 1 conflicts with task 2 and task 5, task 7 is in conflict with task 3, task 5 is in conflict with task 1, task 4, and task 6.

**Privacy conflict matrix:** We can further describe the privacy conflict graph with a privacy conflict matrix, which is an n×n matrix. If there is a privacy conflict between task *i* and task *j*, A[i,j]=1; vice versa A[i,j]=0. The privacy conflict matrix corresponds to the privacy conflict graph in Figure 2 is

(5) $$A=\left[\begin{array}{ccccccc} 0 & 1 & 0 & 0 & 1 & 0 & 0\\ 1 & 0 & 0 & 0 & 0 & 0 & 0\\ 0 & 0 & 0 & 0 & 0 & 0 & 1\\ 0 & 0 & 0 & 0 & 1 & 0 & 0\\ 1 & 0 & 0 & 1 & 0 & 1 & 0\\ 0 & 0 & 0 & 0 & 1 & 0 & 0\\ 0 & 0 & 1 & 0 & 0 & 0 & 0 \end{array}\right].$$

The association privacy of a mobile device is measured by the number of privacy conflicts during task offloading. When $\alpha_{i}=0$, task *i* is executed locally; there is no risk of the association privacy leakage. When $\alpha_{i}\neq 0$, the mobile device chooses to offload task *i* to some edge server for execution. At this time, if a task on the edge server conflicts with task *i*, offloading task *i* to this server may lead to privacy information leakage. We denote the set of tasks that have been offloaded on edge server *j* as $S_{j}$. $n_{j}^{s}$ denotes the number of offloaded tasks on the edge server *j*. $S_{j}\left[K\right]$ denotes the *K*-th task’s sequence number on server *j*. For example, we offload task 3 to server *j* when there is no task present on server *j*. That is, task 3 is the first task on server *j*. Then, $S_{j}\left[1\right]=3$. Thus, the association privacy of a mobile device when offloading task *i* can be expressed as

(6) $$\gamma_{i}=\left\{\begin{array}{cc} \sum_{K}^{n_{j}^{s}}A\left[i,S_{j}\left[K\right]\right], & \mathrm{if}\;\alpha_{i}\neq 0;\\ 0, & \mathrm{if}\;\alpha_{i}=0. \end{array}\right.$$

A smaller value of $\gamma_{i}$ indicates a smaller risk of association privacy leakage and vice versa. Then the association privacy of a mobile device can be expressed as

(7) $$\gamma =\frac{\sum_{i}^{n}\gamma_{i}}{n}.$$

### 3.3. Time Model

Using proxy forwarding causes additional delays. Additional delays increase the completion time of tasks. If the completion time exceeds the maximum deadline of a task, the task will not be completed, which can seriously affect the quality of the user’s experience. Therefore, the completion time of tasks needs to be considered.

Tasks can be executed locally or offloaded to an edge server for execution. When task *i* is executed locally, its completion time is

(8) $$T_{i}^{local}=\frac{c_{i}}{f_{u}}.$$

$f_{u}$ is the computing capacity of the mobile device *u* and $c_{i}$ is denoted as the total number of CPU cycles for accomplishing task *i*.

When task *i* is offloaded to an edge server for execution, task *i* needs to be forwarded through the proxy server. In this case, the completion time of task *i* includes the transmission time from task *i* to proxy server *k*, the transfer time from proxy server *k* to edge server *j*, the execution time of task *i* on edge server *j*, and the return time of the computation result of task *i*. As the amount of data returned is small, we do not consider the backhaul time here. Therefore, the completion time of task *i* is

(9) $$T_{i}^{remote}=\frac{d_{i}}{R_{k}}+\frac{d_{i}}{R_{k,j}}+\frac{c_{i}}{F_{j}}.$$

$F_{j}$ is the computing capacity of the edge server *j*, and $c_{i}$ is denoted as the total number of CPU cycles for accomplishing the task *i*. $R_{k}$ is the transmission rate from mobile device *u* to proxy server *k*, which is calculated as follows:

(10) $$R_{k}=W\log\left(1+\frac{dis{\left(u,P_{k}\right)}^{-l}p}{\sigma^{2}}\right),$$

where *p* is the transmission power of the mobile device *u*, $\sigma^{2}$ is the Gaussian noise, *W* is the bandwidth of the wireless channel and *l* is the path loss factor. $R_{k,j}$ denotes the data transmission rate from proxy server *k* to edge server *j*, which is calculated as follows:

(11) $$R_{k,j}=W\log\left(1+\frac{dis{\left(P_{k},M_{j}\right)}^{-l}p_{k}^{*}}{\sigma^{2}}\right),$$

where $p_{k}^{*}$ is the transmission power of the proxy server *k*. Thus, the completion time of task *i* can be defined as

(12) $$T_{i}=\left\{\begin{array}{c} T_{i}^{\mathrm{local}\;},\;\mathrm{if}\;\alpha_{i}=0;\\ T_{i}^{\mathrm{remote}\;},\;\mathrm{if}\;\alpha_{i}\neq 0. \end{array}\right.$$

The average completion time of mobile device *u*’s tasks is

(13) $$T=\frac{\sum_{i}^{n}T_{i}}{n}.$$

### 3.4. Problem Definition

After establishing all models, we now give the mathematical description of the studied problem in this paper. The problem is formulated as follows:

(14) $$\begin{array}{cc} \min_{\alpha_{i},\beta_{i}}\left(T,\lambda ,\gamma \right) & =\min_{\alpha_{i},\beta_{i}}\left(\frac{\sum_{i}^{n}T_{i}}{n},\frac{\sum_{i}^{n}\left(\lambda_{i}^{k,j}\right)}{n},\frac{\sum_{i}^{n}\gamma_{i}}{n}\right)\\ subject\;to\\ & c1:\beta_{i}\in \left\{0,1,2,\ldots ,N\right\},i\in t,\\ & c2:\alpha_{i}\in \left\{0,1,2,\ldots ,m\right\},i\in t,\\ & c3:T_{i}\leq T_{i}^{\max},i\in t,\\ & c4:\beta_{i}\neq 0,\;\mathrm{if}\;\alpha_{i}\neq 0. \end{array}$$

c1 restricts that a task can be executed locally or first offloaded to one of the proxy servers from 1 to *N* for forwarding. c2 indicates that a task can be executed locally or on one of the edge servers from 1 to *m*. c3 restricts that the completion time of a task cannot exceed its maximum deadline. c4 indicates that a proxy server must be selected when a task needs to be offloaded.

## 4. The Proposed Technique

To solve the above problem, a privacy-preserving task offloading algorithm (PTOA) is designed. In the following, we will describe the algorithm in detail.

### 4.1. Task Classification

Before selecting edge servers for offloading and proxy servers for forwarding tasks, we need to partition all tasks into two classes. If a task can be completed locally within its maximum deadline, the task will be assigned to the completable set $list_{1}$; otherwise, the task will be assigned to the non-completable task set $list_{2}$.

### 4.2. Priority

To better solve privacy conflicts and reduce task completion time, we design two kinds of priorities, one for tasks, and the other for edge servers. The priority for tasks relies on tasks’ privacy conflict level and data size.

**Definition** **1** (Privacy Conflict Level). *Denote $\partial_{i}$ to be the privacy conflict level of task i, and it records the number of tasks that conflict with i. Then we have*

(15) $$\partial_{i}=\sum_{j=1}^{n}A\left[i,j\right],$$

*where n is the total number of tasks.*

After that, we show how to calculate the priority of a task. Denote $\xi_{i}$ to be the priority of task *i*, and it is calculated by

(16) $$\xi_{i}=\frac{c_{i}}{\partial_{i}}.$$

The higher the value of $\xi_{i}$, the higher the priority of task *i*. High-priority tasks have a larger total number of CPU cycles needed to compute and smaller privacy conflict levels.

Next, we will show how to compute the priority of an edge server. Let $\psi_{j}$ be the priority of edge server *j*, and then it is calculated by

(17) $$\psi_{j}=\frac{F_{j}}{dis\left(u,M_{j}\right)}.$$

The higher the value of $\psi_{j}$, the higher the priority of edge server *j*. High-priority servers have more computing capacity and are closer to mobile devices.

### 4.3. The Policy to Select Edge Servers

We prefer to offload a task with higher priority to an edge server with higher priority. Because the task with higher priority has a larger data size and a smaller privacy conflict level, offloading it to the edge server with higher priority can reduce the risk of association privacy leakage and task execution time. The main steps of the policy to select edge servers for tasks is as follows: (1) For the first task with the highest priority denoted by $list_{2}\left[1\right]$ in $list_{2}$, we check if there is any task that conflicts with it on the first edge server with the highest priority denoted by $M_{1}$ in *M*. If the answer is no, then we assign $list_{2}\left[1\right]$ to edge server $M_{1}$; otherwise, we check if there is any task that conflicts with $list_{2}\left[1\right]$ on the second edge server with the second highest priority denoted by $M_{2}$ in *M*. If the answer is no, then we assign $list_{2}\left[1\right]$ to edge server $M_{2}$. Repeat the above operations until we successfully find an edge server for task $list_{2}\left[1\right]$, or we do not successfully assign the task to any edge server after checking all edge servers. For the former, we remove $list_{2}\left[1\right]$ from $list_{2}$ and go on finding an edge server for the next task in $list_{2}$. For the latter, we still keep $list_{2}\left[1\right]$ in $list_{2}$ and continue to search an edge server for the next task in $list_{2}$. (2) We make the same operations to all the rest tasks in $list_{2}$ as we do to $list_{2}\left[1\right]$. (3) Moreover, we make the same operations to all the tasks in $list_{1}$ as we do to $list_{2}\left[1\right]$. (4) After that, if set $list_{2}$ is not empty, for each unassigned task in $list_{2}$, we calculate the number of tasks that have privacy conflicts with it on each server, and assign the task to the edge server with the smallest number of tasks that conflict with the task. If $list_{1}$ is not empty, all unassigned tasks in $list_{1}$ will be processed locally.

### 4.4. Proxy Server Selection Strategy

The choice of a proxy server for a mobile device is important. If the proxy server is close to the mobile device, the approximate location of the mobile device can still be known, thus, causing location privacy leakage. If the proxy server is far from the mobile device, it will cause high latency. For these reasons, a proxy server selection strategy that considers both privacy and latency is designed.

We use $\eta_{i}^{k,j}$ to denote the loss caused by selecting proxy server *k* for forwarding task *i* when it is offloaded to edge server *j* for execution. The loss $\eta_{i}^{k,j}$ is calculated as follows:

(18) $$\begin{array}{cc} \; & \eta_{i}^{k,j}=\mu {\left(\frac{dis(u,M_{j})}{dis(u,P_{k})}\right)}^{2}+\omega \left(\sqrt{dis\left(u,P_{k}\right)+dis\left(P_{k},M_{j}\right)-dis\left(u,M_{j}\right)}\right), \end{array}$$

where μ and ω are weighting factors to balance the effect of location privacy and latency. μ∈[0,1], ω∈[0,1], and μ+ω=1. If task *i* is offloaded to edge server *j*, we select a proxy server with the smallest $\eta_{i}^{k,j}$ for it. Thus, our proposed algorithm can be described as shown in Algorithm 1.
**Algorithm 1** PTOA**Input:** 
Proxy server set *P*, task set *t*, edge server set *M*.**Output:** 
The scheme of selecting edge servers α, the scheme of selecting proxy servers β, privacy conflict matrix *A*, Location of all devices.1:Classify all tasks and store them into sets $list_{1}$ as well as $list_{2}$ according to the rule introduced in Section 4.1.2:Calculate the priorities of tasks and edge servers based on Equations (16) and (17).3:Sorting tasks in $list_{1}$, $list_{2}$ and edge servers in *M* in descending order of their priorities.4:**for**i=1 to $size(list_{2})$ **do**5:    Select edge server for task $list_{2}\left[i\right]$ based on Section 4.3.6:    **if** task $list_{2}\left[i\right]$ successfully selects a server **then**7:        Record the edge server selection policy of this task in α and remove it from $list_{2}$.8:    **else**9:        Retain it in $list_{2}\left[i\right]$.10:    **end if**11:**end for**12:**for**i=1 to $size(list_{1})$ **do**13:    Select edge server for task $list_{1}\left[i\right]$ based on Section 4.3.14:    **if** task $list_{1}\left[i\right]$ successfully selects a server **then**15:        Record the edge server selection policy of this task in α and remove it from $list_{1}$.16:    **else**17:        Retain it in $list_{1}\left[i\right]$.18:    **end if**19:**end for**20:**if**$list_{2}$ is not empty **then**21:    for each unassigned task in $list_{2}$, assign the task to the edge server with the smallest number of tasks that conflict with the task, and record the edge server selection policy of this task in α.22:**end if**23:**if**$list_{1}$ is not empty **then**24:    Select local calculation for the remaining tasks in $list_{1}$.25:**end if**26:**for**i=1 to *n* **do**27:    Select the proxy server with the smallest $\eta_{i,k,j}$ for task *i* according to Equation (18) and record the proxy server selection policy of this task in β.28:**end for**

### 4.5. Time Complexity Analysis

Here we will analyze the time complexity of the PTOA algorithm, which mainly consists of the following time complexities. The time complexity of computing the conflicting level of tasks is $O(n^{2})$. The time complexity of calculating the priority of tasks is O(n). The time complexity of calculating the edge server priority is O(m). The time complexity of sorting task priorities and edge server priorities are O(mlogm) and O(nlogn), respectively. The time complexity of selecting edge servers for tasks is $O(n^{2}\times m)$. The time complexity of selecting proxy servers for tasks is O(n×N). Thus, the time complexity of our proposed PTOA algorithm in the worst case is $O\left(n^{2}\right)+O\left(n\right)+O\left(m\right)+O\left(mlogm\right)+O\left(nlogn\right)+O\left(n^{2}\times m\right)+O\left(n\times N\right)=O\left(n^{2}\times m+nN\right)$.

## 5. Simulation Experiments

In this section, we will evaluate the performance of our proposed algorithm, PTOA, through simulation experiments. First, we will present the specific experiment-related parameter setting. Then, we will display all experimental results and discuss them.

### 5.1. Experimental Parameter Setting

We assume that there are 10 edge servers and 100 proxy servers in the MEC offloading scenario. The locations of mobile device *u*, proxy servers, and edge servers are randomly distributed in a two-dimensional plane with dimensions 1500 m × 1500 m. There is a 25% probability of privacy conflicts between the tasks of the mobile device *u*. The bandwidth of the wireless channel is 5 MHz, the Gaussian noise is $\sigma^{2}=-70$ dbm, and the path loss factor is l=3. The rest parameter settings for experiments are shown in Table 1.

### 5.2. Simulation Results and Analysis

Since there are no researches having the same MEC scenario as the MEC scenario in our work, like many other similar kinds of research, we only use random offloading (Random), all offloading (All), and local computing (Local) as comparison methods. To ensure stability and accuracy, each experiment is repeated 500 times. In each repetition, devices (including mobile devices, proxy servers, and edge servers) location, the data size of tasks, and server-related parameters are randomly generated. The average result of 500 repetitions is taken as the final result of each experiment.

**Figure 3:**
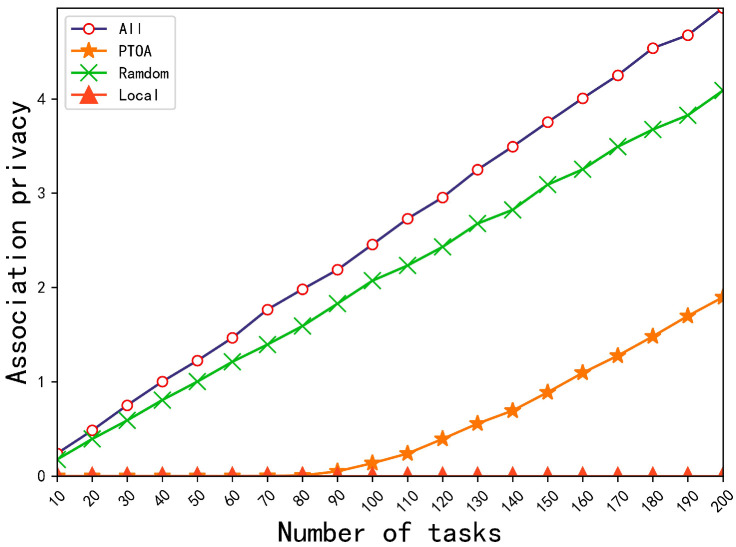
Comparison of the association privacy-preserving ability for four methods, All, PTOA, Random, and Local, with different numbers of tasks.

First, we compare the effect of the number of tasks on the association privacy-preserving ability, the location privacy-preserving ability, and the average completion time of PTOA with baseline methods. Specifically, Figure 3 shows the comparison of the association privacy-preserving ability for four methods, All, PTOA, Random, and Local, under a different number of tasks. It is obvious that local computing (Local) has the best protection for association privacy because there is no risk of association privacy leakage. Our PTOA algorithm has the same protection for association privacy as the Local method when the number of tasks is less than or equal to 80. When the number of tasks goes on increasing, PTOA has lower protection for association privacy. However, our PTOA algorithm is superior to the methods of all offloading (All) and random offloading (Random) in terms of protecting the association’s privacy all the time. Table 2 shows the comparison of the location privacy-preserving ability of PTOA with the methods All and Random with different numbers of tasks. Since there is no risk of location privacy leakage in local computing, we are not making comparisons here. From Table 2, it is observed that our PTOA algorithm outperforms the methods All and Random in protecting location privacy; compared with all offloading and random offloading, the protection of location privacy by our algorithm is separately improved by 43.09% and 33.95% on average. Table 3 shows the comparison of the average completion time of PTOA with the methods All, Random, and Local with a different number of tasks. It can be seen that our PTOA algorithm achieves the smallest average completion time; compared with all offloading, random offloading, and local computing, the average completion time by our algorithm is reduced by 14.06%, 35.57%, and 75.37% on average, respectively.

**Figure 4:**
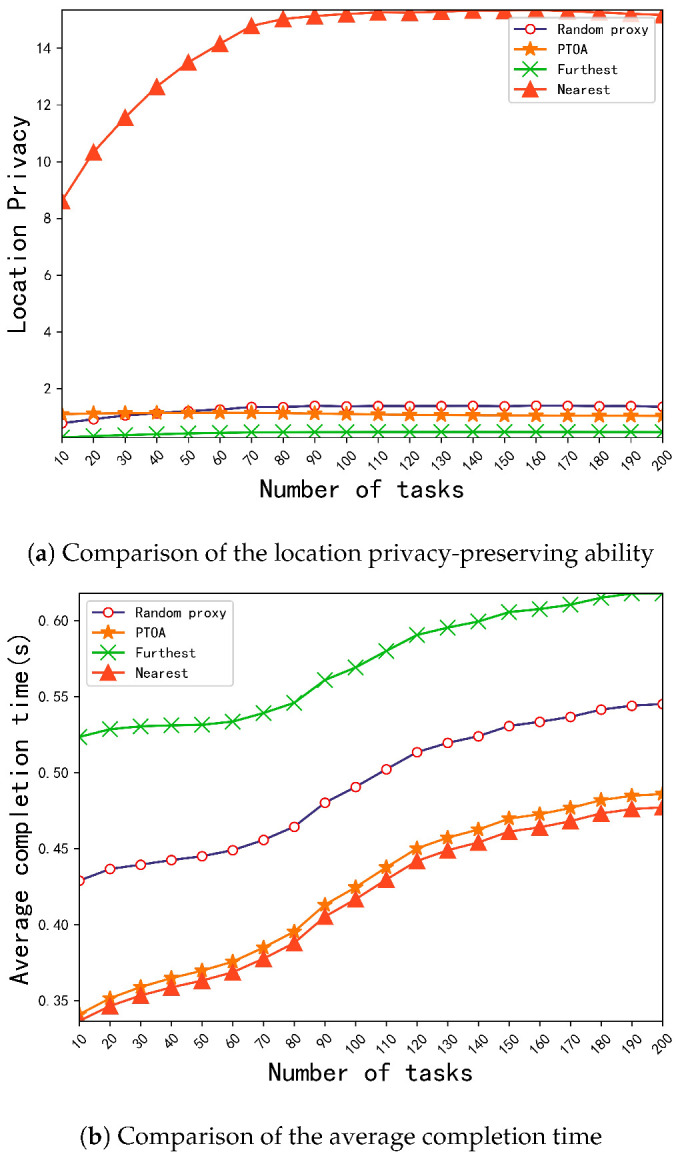
Comparison of the location privacy-preserving ability and average completion time for four different proxy server selection strategies Random proxy, PTOA, Furthest, and Nearest with different numbers of tasks.

Second, we compare the effect of the number of tasks on the location privacy-preserving ability and average completion time of PTOA with three other proxy server selection policies, Random proxy, Furthest, and Nearest, as shown in Figure 4a,b. The Random proxy policy is to choose a proxy server for a mobile device randomly; the Furthest method is to select the proxy server which is the farthest from a mobile device among all proxy servers for the mobile device; the Nearest method is to select the proxy server which is the closest to a mobile device among all proxy servers for the mobile device. Since we do not change the server selection policy and the associated privacy is invariant, we no longer discuss the association privacy. It can be seen from Figure 4a that our PTOA algorithm outperforms the Random proxy and Nearest policies in terms of protecting location privacy. Although PTOA is not as good as the Furthest policy in protecting location privacy, choosing the farthest proxy server increases the average completion time of tasks. It can be seen from Figure 4b that our PTOA algorithm is superior to the Random proxy and Furthest policies in terms of average completion time. PTOA is not as good as the Nearest policy in completion time, but offloading tasks to the proxy server closest to the mobile device increases the risk of location information leakage.

Moreover, considering that the number of proxy servers and the number of edge servers are not fixed in the actual scenario, we compare the effect of a different number of proxy servers and edge servers on the location privacy-preserving ability and average completion time of PTOA with baseline methods.

Table 4 and Table 5 show that the comparison of the location privacy-preserving ability and average completion time of PTOA with the methods All and Random with a different number of proxy servers when the number of tasks is 200, respectively. Since we do not change the server selection policy and the associated privacy is invariant, we no longer discuss the association privacy. It can be seen from Table 4 that our PTOA algorithm is 30.32% and 23.28% averagely higher than all offloading and random offloading in protecting location privacy. At the same time, the variation in the number of proxy servers has a minor impact on our algorithm, and, thus, our PTOA has a better stability. From Table 5, it can be seen that when the number of proxy servers increases, our proposed algorithm always outperforms the Random method in terms of the average completion time. When the number of proxy servers is small, that is, less than 40, our algorithm is not as effective as all offloading. The reason for this is that our algorithm requires a trade-off between privacy protection and latency. When the number of proxy servers is larger than or equal to 40, our algorithm is more effective than the All method. This is because there are more proxy servers to choose from, and our algorithm makes it easy to choose a proxy server that protects privacy without having a great impact on completion time. Averagely, it can be seen from Table 5 that our proposed algorithm can reduce the average completion time by 0.8% and 25.60% compared with all offloading (All) and random offloading (Random).

**Figure 5:**
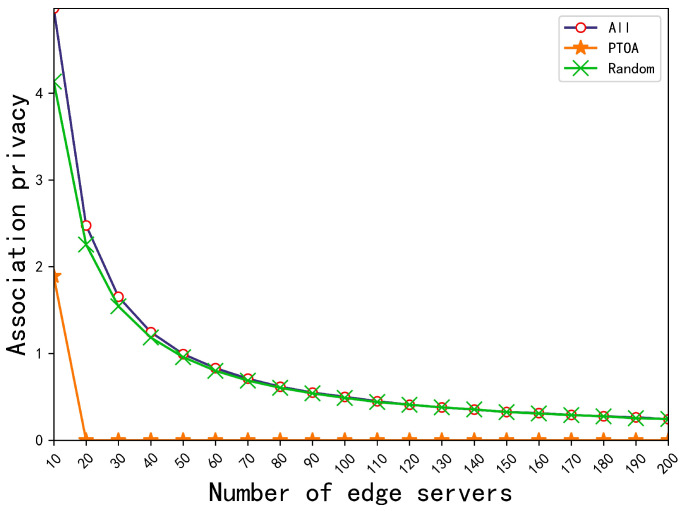
Comparison of the association privacy-preserving ability for three different strategies, All, Random, and PTOA, with a different number of edge servers.

Figure 5 and Table 6 and Table 7 compare the effects of a different number of edge servers on the association privacy-preserving ability, the location privacy-preserving ability, and the average completion time of PTOA with baseline methods when the number of tasks is 200, respectively. It is obvious that our PTOA algorithm is still superior to other methods in aspects of protecting association privacy, protecting location privacy, and reducing latency. It can be seen from Table 6 that the protection ability of our strategy for location privacy is 26.03% and 24.66% higher than that of all offloading and random offloading on average, respectively. It can be seen from Table 7 that our strategy reduces the average completion time by 29.98% and 34.25% on average compared with all offloading and random offloading, respectively.

On the whole, simulated experimental results show that our proposed PTOA algorithm outperforms baseline methods in protecting association privacy, protecting location privacy, and reducing latency.

## 6. Conclusions

In this paper, we studied the privacy protection problem of task offloading in MEC. Location privacy and association privacy during task offloading are considered. MSS proxy forwarding mechanism is used to effectively reduce the risk of location privacy leakage for mobile devices. A privacy-preserving algorithm is designed to select suitable proxy and edge servers for task offloading, to effectively reduce the risk of privacy leakage and minimize the average completion time of tasks. Finally, we demonstrate the effectiveness and stability of our algorithm through simulation experiments. In future work, we will consider resource constraints, energy consumption, and the cost of devices.

## Figures and Tables

**Table 1:** Experimental parameters.

Parameters	Value Range
Mobile device computing capacity: $f_{i}$	1.5 GHz
The total number of CPU cycles for accomplishing the task *i*: $c_{i}$	$3\times 10^{2}$ Megacycles–$6\times 10^{3}$ Megacycles
Mobile device transmission power: *p*	2.5 W
Proxy servers transmission power: $p_{k}^{*}$	5 W
Maximum Deadline $T_{i}^{max}$	1 s
Maximum computing capacity of edge servers: $F_{j}$	5 GHz–20 GHz
Task data volume size: $d_{i}$	1 Mbit–10 Mbit

**Table 2:** Comparison of the location privacy-preserving ability of PTOA with the methods All and Random with different numbers of tasks.

Number of Tasks	All	Random	PTOA	Improvement of PTOA vs. All	Improvement of PTOA vs. Random
10	1.8881	1.6022	1.0987	41.81%	31.43%
20	1.9270	1.6619	1.1226	41.74%	32.45%
30	1.9406	1.6480	1.1347	41.53%	31.15%
40	1.9140	1.6545	1.1496	39.94%	30.52%
50	1.9675	1.6702	1.1521	41.44%	31.02%
60	1.9016	1.6682	1.1584	39.09%	30.56%
70	1.9315	1.6619	1.1586	40.02%	30.29%
80	1.9568	1.6885	1.1468	41.39%	32.08%
90	1.9462	1.6854	1.1268	42.10%	33.14%
100	1.9479	1.6822	1.1164	42.69%	33.63%
110	1.9565	1.6777	1.1005	43.75%	34.40%
120	1.9263	1.6782	1.0970	43.05%	34.64%
130	1.9343	1.6851	1.0840	43.96%	35.67%
140	1.9531	1.6755	1.0712	45.15%	36.07%
150	1.9367	1.6774	1.0664	44.94%	36.42%
160	1.9208	1.6785	1.0629	44.66%	36.68%
170	1.9614	1.6780	1.0526	46.34%	37.27%
180	1.9372	1.6755	1.0511	45.74%	37.27%
190	1.9457	1.6702	1.0480	46.14%	37.26%
200	1.9515	1.6642	1.0454	46.43%	37.18%
Average improvement				43.09%	33.95%

**Table 3:** Comparison of the average completion time of PTOA with the methods All, Random, and Local with different numbers of tasks.

Number of Tasks	All	Random	Local	PTOA	Improvement of PTOA vs. All	Improvement of PTOA vs. Random	Improvement of PTOA vs. Local
10	0.4961	0.6506	1.7284	0.3530	28.84%	45.74%	79.58%
20	0.4971	0.6757	1.7207	0.3542	28.75%	47.58%	79.42%
30	0.4958	0.6638	1.7546	0.3625	26.89%	45.39%	79.34%
40	0.4904	0.6488	1.7046	0.3691	24.72%	43.11%	78.34%
50	0.4945	0.6473	1.7212	0.3739	24.39%	42.23%	78.27%
60	0.4943	0.6712	1.7225	0.3790	23.33%	43.54%	78.00%
70	0.4976	0.6797	1.7565	0.3841	22.81%	43.48%	78.13%
80	0.4973	0.6571	1.7506	0.3959	20.39%	39.75%	77.38%
90	0.4934	0.6544	1.7229	0.4109	16.72%	37.20%	76.15%
100	0.4917	0.6577	1.7271	0.4266	13.24%	35.14%	75.30%
110	0.4954	0.6629	1.7330	0.4426	10.65%	33.23%	74.46%
120	0.4975	0.6616	1.7405	0.4502	9.50%	31.95%	74.13%
130	0.4956	0.6526	1.7272	0.4553	8.14%	30.24%	73.64%
140	0.4964	0.6597	1.7258	0.4677	5.79%	29.11%	72.90%
150	0.4901	0.6550	1.7103	0.4710	3.91%	28.09%	72.46%
160	0.4926	0.6582	1.7109	0.4768	3.20%	27.57%	72.13%
170	0.4929	0.6568	1.7201	0.4809	2.45%	26.78%	72.04%
180	0.4943	0.6559	1.7197	0.4824	2.42%	26.45%	71.95%
190	0.4982	0.6651	1.7195	0.4834	2.97%	27.32%	71.89%
200	0.4922	0.6666	1.7235	0.4832	1.83%	27.50%	71.96%
Average improvement					14.04%	35.57%	75.37%

**Table 4:** Comparison of the location privacy-preserving ability of PTOA with the methods All and Random with different numbers of proxy servers.

Number of Proxy Servers	All	Random	PTOA	Improvement of PTOA vs. All	Improvement of PTOA vs. Random
10	1.5276	1.3864	1.0348	32.26%	25.36%
20	1.4686	1.3370	1.0507	28.46%	21.42%
30	1.4859	1.3523	1.0511	29.26%	22.28%
40	1.6777	1.5153	1.0525	37.26%	30.54%
50	1.5358	1.3963	1.0422	32.14%	25.36%
60	1.4997	1.3560	1.0443	30.37%	22.99%
70	1.3595	1.2405	1.0448	23.15%	15.77%
80	1.3473	1.2233	1.0409	22.74%	14.91%
90	1.5555	1.4094	1.0407	33.10%	26.16%
100	1.4115	1.2802	1.0375	26.50%	18.96%
110	1.3581	1.2397	1.0328	23.95%	16.69%
120	1.4615	1.3192	1.0319	29.39%	21.78%
130	1.4251	1.2978	1.0311	27.64%	20.55%
140	1.4835	1.3509	1.0274	30.74%	23.95%
150	1.4144	1.2853	1.0286	27.27%	19.97%
160	1.5098	1.3732	1.0264	32.02%	25.25%
170	1.8095	1.6423	1.0223	43.51%	37.75%
180	1.4669	1.3310	1.0233	30.24%	23.12%
190	1.4330	1.2989	1.0235	28.58%	21.21%
200	1.6403	1.4874	1.0171	37.99%	31.62%
Average improvement				30.32%	23.28%

**Table 5:** Comparison of the average completion time of PTOA with the methods All and Random under a different number of proxy servers.

Number of Proxy Servers	All	Random	PTOA	Improvement of PTOA vs. All	Improvement of PTOA vs. Random
10	0.4925	0.6588	0.5046	−2.44%	23.41%
20	0.4904	0.6561	0.4973	−1.40%	24.20%
30	0.4932	0.6547	0.4941	−0.17%	24.53%
40	0.4956	0.6611	0.4925	0.63%	25.51%
50	0.4956	0.6592	0.4912	0.88%	25.48%
60	0.4942	0.6613	0.4901	0.82%	25.88%
70	0.4960	0.6610	0.4896	1.28%	25.93%
80	0.4942	0.6566	0.4890	1.05%	25.52%
90	0.4935	0.6585	0.4887	0.97%	25.78%
100	0.4929	0.6576	0.4882	0.96%	25.76%
110	0.4954	0.6562	0.4878	1.54%	25.67%
120	0.4957	0.6630	0.4875	1.65%	26.48%
130	0.4934	0.6556	0.4873	1.25%	25.68%
140	0.4957	0.6654	0.4869	1.77%	26.81%
150	0.4936	0.6565	0.4868	1.39%	25.86%
160	0.4939	0.6608	0.4866	1.48%	26.36%
170	0.4936	0.6547	0.4864	1.46%	25.70%
180	0.4932	0.6553	0.4862	1.42%	25.81%
190	0.4944	0.6553	0.4861	1.68%	25.83%
200	0.4944	0.6557	0.4860	1.72%	25.88%
Average improvement				0.89%	25.60%

**Table 6:** Comparison of the location privacy-preserving ability of PTOA with the methods All and Random with different numbers of edge servers.

Number of Edge Servers	All	Random	PTOA	Improvement of PTOA vs. All	Improvement of PTOA vs. Random
10	1.4492	1.3189	1.0373	28.43%	21.35%
20	1.4222	1.3509	1.1578	18.59%	14.29%
30	1.4169	1.3693	1.1483	18.96%	16.14%
40	1.4320	1.4014	1.1307	21.04%	19.32%
50	1.4269	1.3975	1.1116	22.10%	20.46%
60	1.4197	1.3918	1.1067	22.05%	20.49%
70	1.4184	1.3980	1.0833	23.63%	22.51%
80	1.4222	1.4061	1.0791	24.12%	23.25%
90	1.4190	1.4038	1.0744	24.28%	23.47%
100	1.4209	1.4123	1.0608	25.34%	24.89%
110	1.4184	1.4060	1.0539	25.70%	25.04%
120	1.4235	1.4089	1.0373	27.13%	26.37%
130	1.4260	1.4130	1.0248	28.13%	27.47%
140	1.4282	1.4189	1.0201	28.58%	28.10%
150	1.4191	1.4069	1.0086	28.93%	28.31%
160	1.4254	1.4125	1.0016	29.73%	29.09%
170	1.4259	1.4182	1.0000	29.87%	29.49%
180	1.4236	1.4170	0.9795	31.20%	30.88%
190	1.4224	1.4138	0.9757	31.40%	30.99%
200	1.4205	1.4192	0.9742	31.42%	31.36%
Average improvement				26.03%	24.66%

**Table 7:** Comparison of the average completion time of PTOA with the methods All and Random with different numbers of edge servers.

Number of Edge Servers	All	Random	PTOA	Improvement of PTOA vs. All	Improvement of PTOA vs. Random
10	0.4982	0.6601	0.4919	1.26%	25.48%
20	0.4909	0.5764	0.3802	22.54%	34.04%
30	0.4907	0.5520	0.3582	27.00%	35.11%
40	0.4935	0.5370	0.3518	28.72%	34.49%
50	0.4921	0.5292	0.3468	29.53%	34.47%
60	0.4929	0.5216	0.3417	30.66%	34.48%
70	0.4920	0.5186	0.3401	30.88%	34.43%
80	0.4936	0.5161	0.3366	31.81%	34.78%
90	0.4942	0.5122	0.3373	31.75%	34.15%
100	0.4940	0.5114	0.3329	32.61%	34.90%
110	0.4936	0.5100	0.3330	32.54%	34.71%
120	0.4929	0.5104	0.3305	32.95%	35.25%
130	0.4938	0.5070	0.3311	32.94%	34.69%
140	0.4932	0.5056	0.3303	33.03%	34.67%
150	0.4928	0.5038	0.3283	33.38%	34.83%
160	0.4936	0.5055	0.3300	33.13%	34.70%
170	0.4937	0.5036	0.3276	33.65%	34.95%
180	0.4943	0.5043	0.3284	33.56%	34.87%
190	0.4937	0.5032	0.3274	33.68%	34.93%
200	0.4929	0.5027	0.3254	33.99%	35.27%
Average improvement				29.98%	34.25%

## Data Availability

The datasets generated during and/or analyzed during the current study are available from the corresponding author upon reasonable request.

## References

[1] Ericsson (2020). Ericsson Mobility Report. https://www.ericsson.com.

[2] Aazam M., Zeadally S., Harras K.A. (2018). Offloading in fog computing for IoT: Review, enabling technologies, and research opportunities. Future Gener. Comput. Syst..

[3] Mao Y., You C., Zhang J., Huang K., Letaief K.B. (2017). A Survey on Mobile Edge Computing: The Communication Perspective. IEEE Commun. Surv. Tutor..

[4] Feng C., Han P., Zhang X., Yang B., Liu Y., Guo L. (2022). Computation offloading in mobile edge computing networks: A survey. J. Netw. Comput. Appl..

[5] Huda S.A., Moh S. (2022). Survey on computation offloading in UAV-Enabled mobile edge computing. J. Netw. Comput. Appl..

[6] Haibeh L.A., Yagoub M.C.E., Jarray A. (2022). A Survey on Mobile Edge Computing Infrastructure: Design, Resource Management, and Optimization Approaches. IEEE Access.

[7] Gia T.A.N., Jiang M., Rahmani A.M., Westerlund T., Liljeberg P., Tenhunen H. Fog Computing in Healthcare Internet of Things: A Case Study on ECG Feature Extraction. Proceedings of the 2015 IEEE International Conference on Computer and Information Technology; Ubiquitous Computing and Communications; Dependable, Autonomic and Secure Computing; Pervasive Intelligence and Computing.

[8] Li T., He X., Jiang S., Liu J. (2022). A survey of privacy-preserving offloading methods in mobile-edge computing. J. Netw. Comput. Appl..

[9] Wang W., Ge S., Zhou X. Location-Privacy-Aware Service Migration in Mobile Edge Computing. Proceedings of the 2020 IEEE Wireless Communications and Networking Conference (WCNC).

[10] Li T., Liu H., Liang J., Zhang H., Geng L., Liu Y. Privacy-Aware Online Task Offloading for Mobile-Edge Computing. Proceedings of the WASA.

[11] Sun Y., Li N., Tao X. Privacy Preserved Secure Offloading in the Multi-access Edge Computing Network. Proceedings of the 2021 IEEE Wireless Communications and Networking Conference Workshops (WCNCW).

[12] Zhang H., Wang Y., Du X., Guizani M. Preserving Location Privacy in Mobile Edge Computing. Proceedings of the ICC 2019—2019 IEEE International Conference on Communications (ICC).

[13] Peng K., Liu P., Huang T. A Privacy-aware Computation Offloading Method for Virtual Reality Application. Proceedings of the CIKM Workshops.

[14] Xu X., Xue Y., Qi L., Yuan Y., Zhang X., Umer T., Wan S. (2019). An edge computing-enabled computation offloading method with privacy preservation for internet of connected vehicles. Future Gener. Comput. Syst..

[15] Liu X., Xu X., Yuan Y., Zhang X., Dou W. Energy-Efficient Computation Offloading with Privacy Preservation for Edge Computing-Enabled 5G Networks. Proceedings of the 2019 International Conference on Internet of Things (iThings) and IEEE Green Computing and Communications (GreenCom) and IEEE Cyber, Physical and Social Computing (CPSCom) and IEEE Smart Data (SmartData).

[16] Xu Z., Gu R., Huang T., Xiang H., Zhang X., Qi L., Xu X. (2018). An IoT-Oriented Offloading Method with Privacy Preservation for Cloudlet-Enabled Wireless Metropolitan Area Networks. Sensors.

[17] Ma W., Mashayekhy L. Privacy-by-Design Distributed Offloading for Vehicular Edge Computing. Proceedings of the 12th IEEE/ACM International Conference on Utility and Cloud Computing.

[18] Xu X., He C., Xu Z., Qi L., Wan S., Bhuiyan M.Z.A. (2020). Joint Optimization of Offloading Utility and Privacy for Edge Computing Enabled IoT. IEEE Internet Things J..

[19] Xu X., Tang B., Jiang G., Liu X., Xue Y., Yuan Y. Privacy-Aware Data Offloading for Mobile Devices in Edge Computing. Proceedings of the 2019 International Conference on Internet of Things (iThings) and IEEE Green Computing and Communications (GreenCom) and IEEE Cyber, Physical and Social Computing (CPSCom) and IEEE Smart Data (SmartData).

[20] Yao Y., Wang Z., Zhou P. (2020). Privacy-preserving and energy efficient task offloading for collaborative mobile computing in IoT: An ADMM approach. Comput. Secur..

[21] Li K., Han X., Yang Y., Wang S., hua Shi R., Li J. A Novel Edge Computing Offloading and Privacy-preserving Scheme for Energy Internet. Proceedings of the 2021 IEEE 5th International Conference on Cryptography, Security and Privacy (CSP).

[22] Schlegel R., Kumar S., Rosnes E., i Amat A.G. (2022). Privacy-Preserving Coded Mobile Edge Computing for Low-Latency Distributed Inference. IEEE J. Sel. Areas Commun..

[23] Zhang P., Durresi M., Durresi A. (2018). Multi-access edge computing aided mobility for privacy protection in Internet of Things. Computing.

[24] Chen X., Jiao L., Li W., Fu X. (2016). Efficient Multi-User Computation Offloading for Mobile-Edge Cloud Computing. IEEE/ACM Trans. Netw..

[25] Labidi W., Sarkiss M., Kamoun M.A. Joint multi-user resource scheduling and computation offloading in small cell networks. Proceedings of the 2015 IEEE 11th International Conference on Wireless and Mobile Computing, Networking and Communications (WiMob).

[26] Guo F., Zhang H., Ji H., Li X., Leung V.C.M. (2018). An Efficient Computation Offloading Management Scheme in the Densely Deployed Small Cell Networks With Mobile Edge Computing. IEEE/ACM Trans. Netw..

[27] Mao Y., Zhang J., Letaief K.B. (2016). Dynamic Computation Offloading for Mobile-Edge Computing With Energy Harvesting Devices. IEEE J. Sel. Areas Commun..

[28] Zhou S., Jadoon W. (2021). Jointly Optimizing Offloading Decision and Bandwidth Allocation with Energy Constraint in Mobile Edge Computing Environment. Computing.

[29] Liu J., Mao Y., Zhang J., Letaief K.B. Delay-optimal computation task scheduling for mobile-edge computing systems. Proceedings of the 2016 IEEE International Symposium on Information Theory (ISIT).

[30] Zhang P., Durresi M., Durresi A. Network Location Privacy Protection with Multi-access Edge Computing. Proceedings of the AINA.

